# Coupling between Grand cycles and Events in Earth’s climate during the past 115 million years

**DOI:** 10.1038/s41598-018-36509-7

**Published:** 2019-01-23

**Authors:** Slah Boulila

**Affiliations:** 1 Sorbonne Université, CNRS, Institut des Sciences de la Terre Paris, ISTeP, F-75005 Paris, France; 2 ASD/IMCCE, CNRS-UMR8028, Observatoire de Paris, PSL University, Sorbonne Université, 77 Avenue Denfert-Rochereau, 75014 Paris, France

## Abstract

Geological sediment archives document a rich periodic series of astronomically driven climate, but record also abrupt, severe climatic changes called events, the multi-Myr boundary conditions of which have generally been ascribed to acyclic processes from Earth’s interior dynamics. These events have rarely been considered together within extended time series for potential correlation with long-term (multi-million year, Myr) cycling. Here I show a coupling between events and multi-Myr cycles in a temperature and ice-volume climatic proxy of the geological past 115 Myr. I use Cenozoic through middle Cretaceous climatic variations, as recorded in benthic foraminifera δ^18^O, to highlight prominent ~9 and ~36 Myr cyclicities. These cyclicities were previously attributed either to astronomical or tectonic variations. In particular, I point out that most of the well-known events during the past 115 Myr geological interval occur during extremes in the ~9 and ~36 Myr cycling. One exception is the early Cenozoic hyperthermal events including the salient Paleocene-Eocene Thermal Maximum (~56 Ma), which do not match extremes in long-period cyclicities, but to inflection point of these cycles. Specific focus on climatic events, as inferred from δ^18^O proxy, suggest that some “events”, marked by gradual trends within the ~9 and ~36 Myr cycle extremes, would principally be paced by long-term cycling, while “events”, recorded as abrupt δ^18^O changes nearby cycle extremes, would be rather induced by acyclic processes. The connection between cyclic and acyclic processes, as triggers or feedbacks, is very likely. Such link between cycling and events in Earth’s past climate provides insight into celestial dynamics governing perturbations in Earth’s surface systems, but also the potential connection between external and Earth’s interior processes.

## Introduction

Earth’s past climate has varied quasi-periodically from hundred to billion years, and these variations were driven by celestial dynamics over a large frequency band^1–3^. In particular, Earth’s climate during the Cenozoic-Late Cretaceous (past 115 million years, Ma) experienced long-term (multi-million year, multi-Myr) changes punctuated by severe, short-lived events^4–8^. The multi-Myr climatic variations have often been considered irregular (acyclic) in nature on the very long term^9^. This has led to the hypothesis that these changes were mainly driven by Earth’s interior dynamics and plate tectonic motions assuming that these processes are acyclic in nature^9^, although more recent studies point to the cyclic behavior of large plate tectonic motions^10–16^. The climatic events have been intensively investigated in terms of their heavy impacts and consequences on Earth’s superficial systems, in particular the resulting profound modifications in ocean and terrestrial environments, expressed as perturbations in the sedimentary biogeochemical cycles^4,5^. Delineating the cyclic versus acyclic nature of multi-Myr climatic variations and their potential link with the climatic events is of paramount importance in deciphering the origin of events and thus their causal mechanisms. However, the study of multi-Myr climatic variations requires the acquisition of high-resolution data covering several million years, hence hampering the characterization of long-term cycling in previous studies^17–19^. New advancements in laboratory analytical techniques together with large collaborative projects allow today the availability of a highly resolved 115-Myr-long climate record from sedimentary benthic foraminifera δ^18^O (see Methods). The δ^18^O record indicates variations in deep-sea temperature as well as changes in ice volume^4,7,8^.

## Results

**Figure 1:**
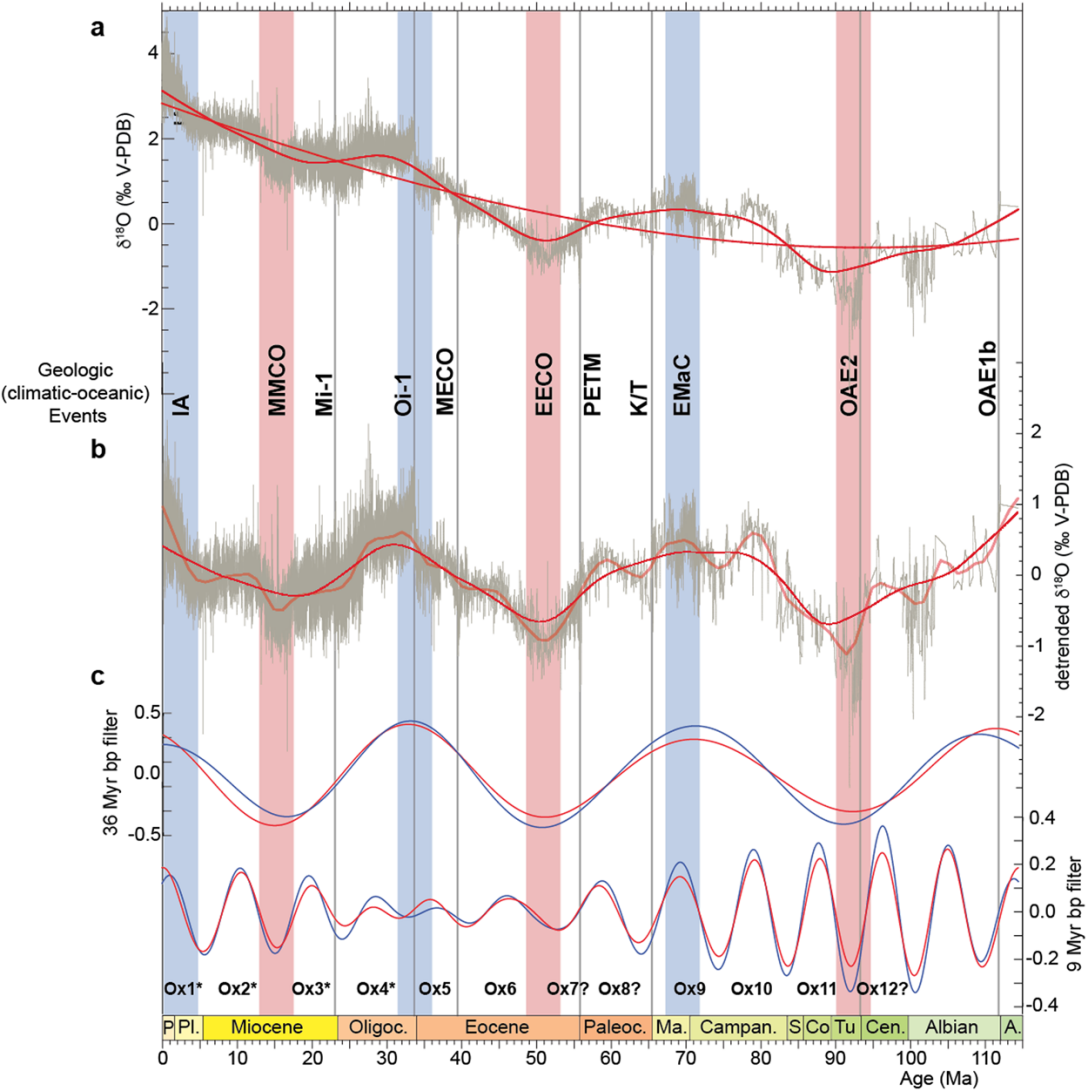
Time-series analysis of benthic foraminifera δ^18^O data of the past 115 Ma (see Methods). **(a)** The raw data with two smoothing fits: a third-order polynomial fit and a 14% weighted-average fit. **(b)** Detrended δ^18^O data (third-order polynomial fit shown in ‘a’ is removed) with two smoothing fits: a 14% weighted-average fit to highlight the 36 Myr cycles, and a 4% weighted-average fit to highlight both the ~9 and ~36 Myr cycles. The well known climatic events are shown: IA: Pliocene-Pleistocenbe Ice Age, MMCO: Mid-Miocene Climatic Optimum, Mi-1 and Oi-1: Oligocene-Miocene and Eocene-Oligocene major glacial events, MECO: Mid-Eocene Climatic Optimum, EECO: Early Eocene Climatic Optimum, PETM: Paleocene-Eocene Thermal Maximum, K/T: Cretaceous/Tertiary boundary, EMaC: Early Maastrichtian Cooling episode, OAE2 and OAE1b: Oceanic Anoxic Events. **(c)** Bandpass filtering of δ^18^O data: 36 Myr cycle band (0.03 ±0.01 cycles/Myr) and 9 Myr cycle band (0.11 ±0.03 cycles/Myr). Ages, P: Pleistocene, Pl.: Pliocene, Oligoc.: Oligocene, Paleoc.: Paleocene, Ma.: Maastrichtian, Campan.: Campanian, S: Santonian, Co: Coniacian, Tu.: Turonian, Cen.: Cenomanian, A.: Aptian. Vertical shaded bars are extrema (minima in red and maxima in blue) of the ~36 Myr δ^18^O cycles, which coincide with the most known thermal and cooling episodes. Also indicated climatic events (vertical grey lines), some of them matching extrema in the ~9 Myr cycles. Ox1 to Ox12 are the interpreted ~9 Myr δ^18^O cycles. Ox1, Ox2, Ox3,… to designate ~9 Myr δ^18^O oscillations 1, 2, 3,… Ox for oxygen (δ^18^O), and increasing numbers indicate the older oscillations. This is used by correlation with Cb1, Cb2, Cb3,… that designate the ~9 Myr δ^13^C oscillations 1, 2, 3,… (Supplementary Figure S1) as in Boulila *et al*.^6^, Cb. for carbon (δ^13^C), and increasing numbers indicate older cycles. Ox1 to Ox4 indicated by asterisks, correlate with their equivalents Cb1 to Cb4 in δ^13^C record (ref.^6^, Supplementary Figure S1). Ox7 and Ox8 indicated by question marks include one oscillation, equivalent to two ~9 Myr oscillations in the δ^13^C record (see text for discussion, and Supplementary Figure S2), Ox12, shown with a question mark, is a poorly constrained cycle, due to low-resolution data within this time interval.

**Figure 2:**
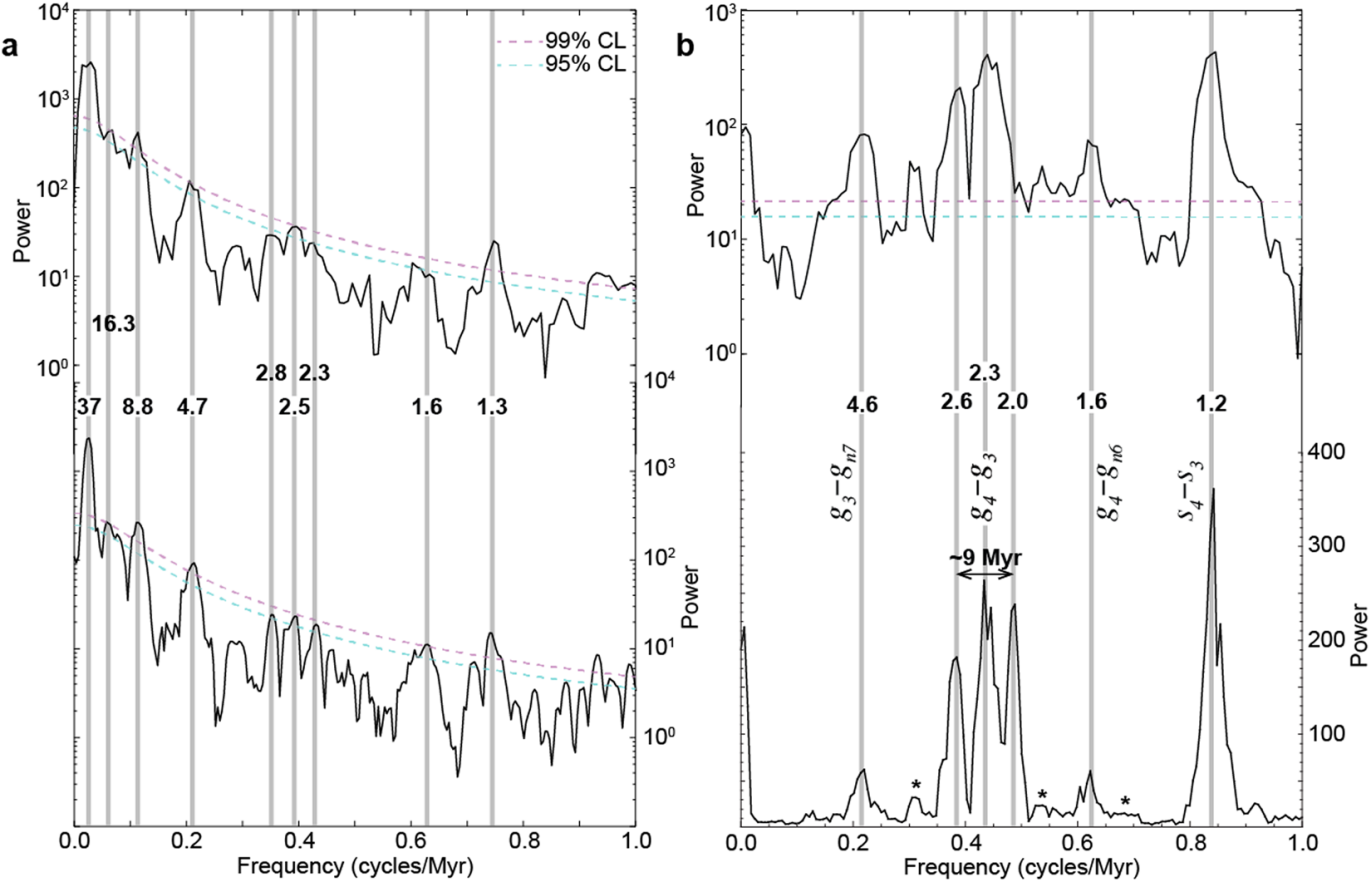
2π-MTM power spectra of benthic foraminifera δ^18^O data of the interval from 0 to 115 Ma (see Methods) and of its time-equivalent astronomical variations. **(a)** Upper panel: Spectrum of the detrended δ^18^O data (50% weighted average of the series is removed), lower panel: Spectrum of 1x zero padded δ^18^O after the detrend (50% weighted average of the series is removed). Significant spectral periods are shown across vertical grey bars. **(b)** Upper panel: Spectrum of lowpass filtered (>1 Myr), composite astronomical time series of the interval 0 to 115 Ma (see Methods), the power axis is a logarithmic scale for comparison with spectra in ‘a’, lower panel: Spectrum of the same astronomical time series (power in linear scale) but over the interval from 0 to 146 Ma (Cenozoic and Cretaceous time). The most prominent astronomical periods and origins of some of them are shown across vertical grey bars, periods indicated by asterisk may represent minor components. Note that the ~9 Myr δ^18^O cyclicity originates from the interfering astronomical terms 2 and 2.6 Myr (see Supplementary Figure S3). The 16.3 Myr δ^18^O period may correspond to a harmonic or may originate from the couple interfering terms 2 Myr vs 2.3 Myr and 2.3 Myr vs 2.6 Myr.

**Figure 3:**
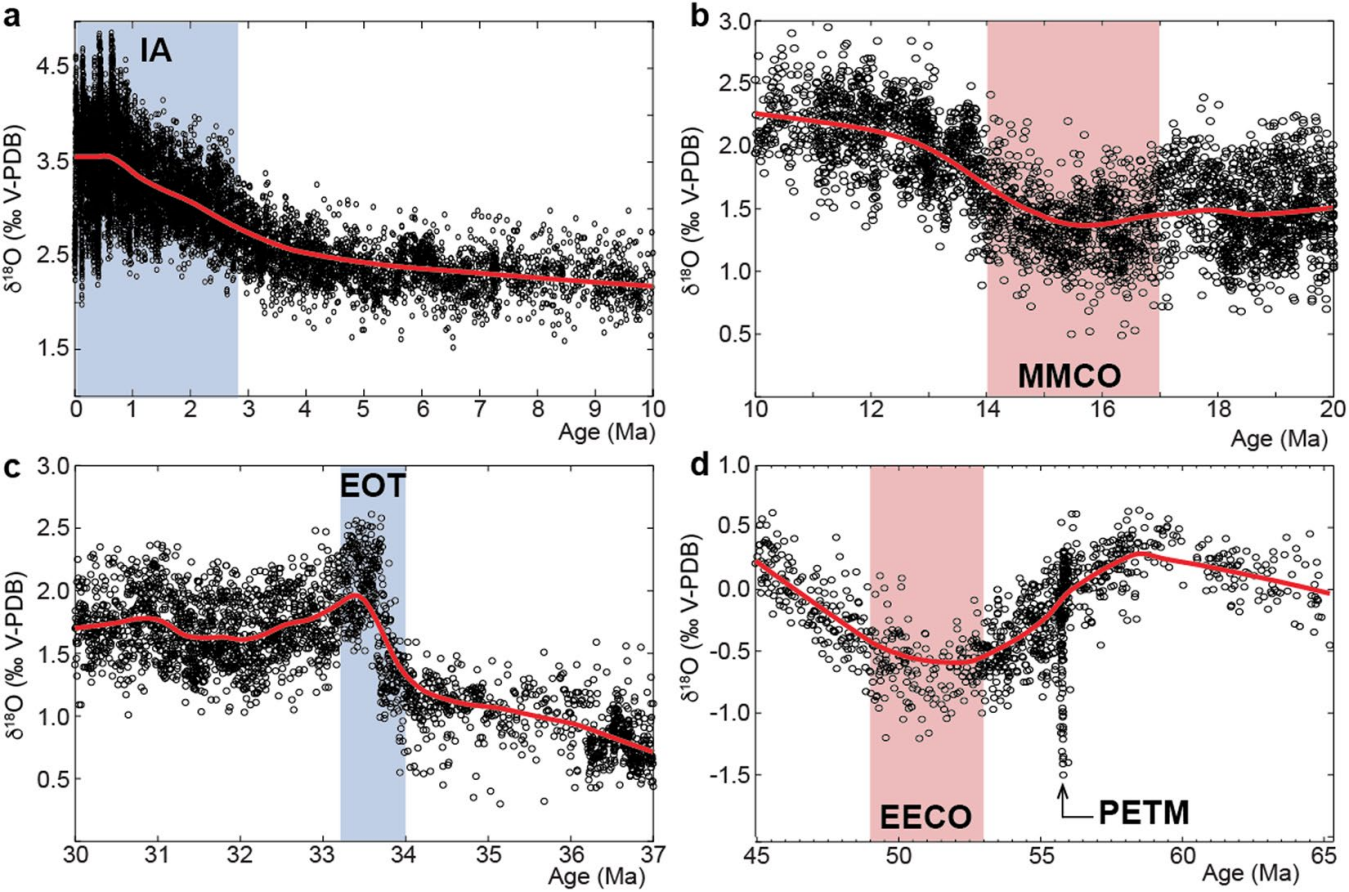
Expanded views of climatic events and phases within the ~36 Myr cycle extrema. The term ‘phase’ opposes to the term ‘event’: ‘event’ indicates abrupt, severe change, whereas ‘phase’ indicates gradual/progressive trend in climate change that could reach an optimum (see below). I also used the term ‘episode’ to designate either event or phase. **(a)** The gradual entry of the Earth into the Pliocene-Pleistocene ice age (IA) as expressed in the trend (40% weighted average). Note that there is no trend during the 100-kyr-cycle dominated climate of the past ca. 800 ka. **(b)** Gradual variations within the Mid-Miocene Climatic Optimum, MMCO (35% weighted average). **(c)** Abrupt change within the Eocene-Oligocene transition (EOT) including the Oi-1 glacial event (15% weighted average). **(d)** Gradual variations within the Early Eocene Climatic Optimum, EECO (35% weighted average). Note that the Paleocene-Eocene Thermal Maximum, PETM, corresponds to a deviation from the long-term cycling within the EECO, and is the most abrupt, severe event in the Cenozoic era.

Time-series analysis of δ^18^O data shows two prominent cyclicities of ~9 and ~36 Myr (Figs 1 and 2). Other low-frequency cyclicities are also present, in particular, 1.3 Myr, 1.6 Myr, a triplet peaks of 2.3, 2.5 and 2.8 Myr, and a 4.7 Myr peak (Fig. 2a), which are close to Milankovitch astronomical periods (Fig. 2b). The 1.3 Myr corresponds to the 1.2 Myr obliquity modulation term. The 1.6 Myr matches the eccentricity term. The triplet corresponds to combined eccentricity and obliquity terms of 2, 2.3 and 2.6 Myr (Fig. 3 and Supplementary Figure S3). Finally, the 4.7 Myr is close to the 4.6 Myr eccentricity peak. Even though spectrally well detected and strikingly close to Milankovitch frequency band (Fig. 3) the periods from 1 to 5 Myr could be altered by the stacking process of the data. Data from single sites document with high fidelity some of these periods^20–32^. In contrast, longer periods (>5 Myr) are notably preserved in the compiled δ^18^O data. In the present study, I focus on the prominent cyclicities of ~9 and ~36 Myr, and their possible link with the geologically well known climatic events. Another potential low-frequency (16 to 18 Myr) cycle could also be seen in the δ^18^O power spectra (Fig. 3), but will not be discussed below.

## Discussion

The ~9 Myr cyclicity was previously detected in carbon-cycle variations^6^. Comparison of δ^18^O and δ^13^C data shows a coupling between climate and carbon cycle at the ~9 Myr cycle band, especially during icehouse, i.e. the past 34 Ma (Supplementary Figure S1). A strong decoupling between them is remarkably noted within the interval from 50 to 65 Ma as follows. While the δ^13^C document the two strongest ~9 Myr cycles Cb7 and Cb8 of the early Cenozoic^6^, the δ^18^O has exceptionally only one oscillation (Fig. 3d, see also Supplementary Figure S2).

The origin of ~9 Myr cyclicity has previously been attributed to the modulation of 2.4 Myr eccentricity cycle band^6^. Here I investigate additional statistical tests to support the modulation at the 2.4 Myr cycle band, but with possible contribution from both eccentricity and obliquity terms (Fig. 2b, and Supplementary Figures S3 and S4). Prominent ~9 Myr geological oscillations have been detected in carbon-cycle and sedimentological proxies of Cenozoic and Mesozoic strata^6,33–35^.

Interestingly, I note four ~9 Myr oscillations grouped in each ~36 Myr cycle (Fig. 1b,c), suggesting a relationship between these two climatic cyclicities. Previous studies ascribed the ~36 Myr climatic variability to another dimension of astronomical forcing^36,37^. In particular, the ~36 Myr period has been attributed to the vertical passage of the Solar System through the Galactic midplane that modulates the flux of galactic cosmic rays (GCR) on Earth^36^. Despite our limited knowledge on the gravitational potential of the Galaxy, there is some consensus that the Solar System vertically oscillates across the Galactic midplane, with a half-period of about 36 Myr^16,38–40^. This effective 36 Myr half-period corresponds to the motion of the Solar System when it moves down- and upwards the galactic midplane. This would induce significant change in GCR flux on Earth, especially when crossing the galactic midplane. This would favor the formation of cloud layer from the incidental GCR flux on Earth, hence resulting in variations of Earth’s albedo and the consequent climate change^16,36,41,42^. Yet, the impact of cosmic ray on climate remains a controversial subject^43^. Another possible hypothesis is that Earth’s interior dynamics may resonate every 36 Myr inducing global climate change, although recent studies pointed to different periodicities of 25 to 50 Myr^13,14^ and 56 Myr^15^. A combined effect from the aforcited mechanisms on climate is presumably^44^. The most intriguing result in time-series analysis of δ^18^O climatic proxy is the record of both ~9 and ~36 Myr oscillations, sharing very likely the same forcing process. While the ~9 Myr periodicity may represent a Milankovitch origin, the ~36 periodicity could be forced by solar system vertical motion, that could in turn modulate Earth’s incident (Milankovitch) insolation, or may correspond to a not resolved Milankovitch band. Although I draw attention to climate and astronomy linkage at the scale of ~9 and ~36 Myr periods, we must caution that the effect of tectonics on climate at this timescale remains well plausible^16^.

Along climate-proxy variations in the Cenozoic and Mesozoic eras (past 0–250 Ma) were recognized and widely studied a number of climatic events^4,5,8^. The studied interval includes some of them (Fig. 1b). Features and nature of Earth’s system responses to these perturbations differ from one event to another^5^, however, tectonic-volcanic mechanisms were thought to be the principal, common cause for most of the events^4^.

Here, I show that most of Cenozoic–Cretaceous climatic events and phases (see Fig. 3 for definition of ‘event’ versus ‘phase’) during the past 115 Ma fall into extremes in amplitudes of the ~9 and ~36 Myr cyclicities (Fig. 1).

For instance, the so-called Oi-1 and Mi-1 glacial episodes are close to the ~9 Myr cycle extremes (Ox4, Fig. 1c). The Mid-Miocene Climatic Optimum (MMCO), which represents a warming phase, bounds ~9 Myr Ox2 and Ox3 δ^18^O oscillations (see Fig. 1 caption for ‘Ox’ cycle numbering). Importantly, some of the events coincide with extremes in the ~36 Myr cycling, thus, they would be with greater magnitudes (Fig. 1b). The three cooling/glacial episodes at Pliocene-Pleistocene Ice Age (IA), Eocene-Oligocene glacial event (Oi-1) and Early Maastrichtian Cooling phase (EMaC) correspond to maxima of the ~36 Myr δ^18^O cycles. The two warming phases of Mid-Miocene Climatic Optimum (MMCO) and Early Eocene Climatic Optimum (EECO) coincide with the ~36 Myr cycle minima. Finally, the globally recognized Oceanic Anoxic Event OAE-2 occurs around a minimum of the ~36 Myr δ^18^O cyclicity. In contrast, the early Cenozoic hyperthermals including the most pronounced Paleocene-Eocene Thermal Maximum (PETM) event do not match extremes in the long-term cycling. The relative variance of the ~36 Myr δ^18^O cyclicity is more than three times higher than that of the ~9 Myr cyclicity. This implies that events (and phases) matching only extremes in the ~9 Myr cycles are with lesser intensities compared to events (and phases) in relation with the ~36 Myr oscillations. However, Earth’s climate response to the forcing processes at the ~9 and ~36 Myr cycle bands from astronomy, for example, could not be direct, because insolation (or GCR) change at these very longer timescales should be weak (see below).

The most striking result is the match between ~9 and ~36 Myr cycle extremes and most of the Cenozoic-Cretaceous events. This result hints at a coupling between Earth’s climate cycling and perturbations. The ~9 and ~36 Myr cyclicities could have an astronomical and/or a tectonic origin (see above). The events have been postulated to be caused by tectonics, volcanism, pCO_2_ trend, etc.

I suggest that among the events in relation with long-period cyclicities some of them may have been mainly related to cyclic process, others may have been the result of combined effects of acyclic and cyclic processes. The IA, MMCO, EECO and possibly EMaC^4,8^ show gradual variations (Figs 1 and 3), thus they may mainly be the result of a cyclic forcing. The Oi-1 and OAE-2^4,5^ show abrupt changes (Figs 1 and 3), thus they may occur acyclically, but could also be triggered by a cyclic mechanism. Large-scale (multi-Myr) Milankovitch astronomical forcing would induce negligible changes in insolation (or GCR) budget on Earth^6^, hence a nonlinear mechanism is required to explain the match between the long-period astroclimatic cycles and events. Energy transfer process from higher to lower frequency driving forces has previously been suggested^6^, but this process cannot argue the match between the cycles and events. An astronomically forced gravitational distortion of Earth’s viscose mantle in boundary conditions may explain the potential link between cycle extremes and events at the multi-Myr timescales. Astronomical control of gravitational deformation (e.g., dynamical ellipticity)^45^ of the mantle at boundary conditions would result in intensification of mantle convection, and the occurrence of climate events, which have been argued to be paced by processes from Earth’s interior dynamics^5,44^. Also, feedback responses of Earth’s interior dynamics to astronomically driven climate and Earth’s surface processes have equally been suggested even at the shorter Milankovitch timescales^46,47^. For instance, glacial cycles have been correlated to oceanic crust production^47,48^. Astroclimatically paced deglaciations would promote mantle decompression, hence increase magma production and thus volcanic eruptions^44,46^.

## Conclusions and Perspectives

In summary, the detection of a potential link between long-period climatic cyclicities (driven by astronomy and/or tectonics) and events provides insights into multi-disciplinary studies involving acyclic versus cyclic nature of Earth’s interior dynamics, astronomical forcing at multi-Myr timescales, and the possible interaction between astronomical and tectonic forcings. The correspondence between very long climate cycle extremes and events during the mid-Cretaceous to Cenozoic time interval suggests a highly nonlinear mechanism response, if the events were responding to insolation, such as astronomical pacing, at boundary conditions, of gravitational perturbation of the Earth’s spin axis and shape. This may, in turn, affect the gravitational deformation of the mantle, stimulate mantle convection and the dynamic topography, hence the occurrence of geodynamically paced climatic events. Cyclic tectonic forcing at the 9 and 36 Myr cycle bands, without evoking the nonlinear astronomical driving force, remains equally plausible.

Future studies should focus on the cyclic vs acyclic nature of Earth’s interior dynamics (tectonics, volcanism,…), at ten to tens of Myr, and likely on how could Earth’s interior dynamics vary or interact with climate^44–51^, and its potential link with the recognized climatic events. Other potential questions remain open, e.g., what would be the paleoenvironmental implications of the strong decoupling, at the 9 Myr oscillations, between climate and carbon cycles during the Paleocene-Eocene extreme greenhouse? Future studies should also focus on the origin of the ~36 Myr climatic cyclicity, by exploring the hypothesis of the possible link between GCR driven climates from vertical motion of the solar system, and insolation (Milankovitch) forced climates.

## Methods

I used compiled benthic foraminifera δ^13^C and δ^18^O data from more than 40 deep-sea cores of the Deep Sea Drilling Project and Ocean Drilling Program^4,7,8^. These data spanning the Cenozoic to the middle-Late Cretaceous, i.e. the past ~115 Ma (Fig. 1a), indicate global variations in climate and carbon cycle, including the well known climatic events^4^. The Cenozoic compilation is from Cramer *et al*.^7^, and the Cretaceous compilation is from Friedrich *et al*.^8^. Although the resolution of the stacked data would distort the record of high-frequency cyclicities, the low-frequency, multi-Myr, if present would emerge in the compiled records^6^. Here I performed time-series analysis on the compiled δ^18^O record to seek for multi-Myr cycling in climate; comparision with long-term carbon cycling^6^ was also carried out (Supplementary Figure S1).

I applied spectral analysis to the δ^18^O data (Fig. 2a) and to long-period (>1 Myr) astronomical variations (Fig. 2b). I generated long-term astronomical variations by extracting amplitude maxima in short eccentricity and amplitude maxima in high-frequency obliquity time series^52,53^, then summing them after standardization, finally I lowpass filtered periods longer than 1 Myr. The astronomical variations from amplitude maxima of the signals are susceptible to detect lower frequencies, as the conventional amplitude modulation technique. I applied this procedure to the interval from 0 to 115 Ma (Fig. 2b, upper spectrum), and to the interval from 0 to 146 Ma (Cenozoic and Cretaceous time, Fig. 2b, lower spectrum). For spectral analysis, I used the multi-taper method (MTM) and the robust red noise as implemented in the ‘astrochron’ R packages^54^. MTM spectral analysis was conducted, with three 2π tapers (Fig. 2), on the linearly resampled (5 kyr) δ^18^O data. To precisely determine the long periods of 9 and 36 Myr I additionally applied 1x zero padding to the δ^18^O time series prior spectral analysis (Fig. 2a). I carried out smoothing and fitting long-term δ^18^O variations using both polynomial method and the Lowess weighted average method (Fig. 1). For the detailed study of δ^18^O record (expanded views in Fig. 3), I have rather performed only the weighted average method on the uneven spaced raw data.

Bandpass filtering was conducted using conjointly the Taner filter, which is characterized by a steep stopband, and the Gaussian filter which has a gentle stopband. Lowpass filtering was conducted using the Taner filter. Filtering δ^18^O signal was applied to the 5 kyr linearly resampled (5 kyr) data, while Lowess weighted average smoothing was applied to the raw data. Finally, in order to highlight the amplitude modulation of the 2.4 Myr cycle band by the ~9 Myr cycles in the astronomical variations, I used evolutive harmonic analysis (EHA) with a function computing a running periodogram of a uniformly sampled time series using FFTs of zero-padded segments, and normalized to the highest amplitudes (Supplementary Figure 3).

## Electronic supplementary material

Supplementary information on time-series analysis
