# Supplementary material for: Coupling between Grand cycles and Events in Earth’s climate during the past 115 million years
Source: Sci Rep. 2019 Jan 23;9:327. doi: 10.1038/s41598-018-36509-7 (PMC6344641; doi:10.1038/s41598-018-36509-7)
Supplement: Supplementary file 1 — Supplementary information on time-series analysis [file 41598_2018_36509_MOESM1_ESM.doc]

**Supplementary Information on:**

**Coupling between Grand cycles and Events in Earth’s climate during the past 115 million years**

**Slah Boulila**

The Supplementary Information contains four figures S1-S4. Figures S1 and S2 show a correlation at the 9 Myr cycle band between climate and carbon-cycle variations from two different benthic foraminifera δ^18^O and δ^13^C compilations of Zachos et al. (2001, 2008) illustrated in Fig. S1, and Cramer et al. (2009) and Friedrich et al. (2012) illustrated in Fig. S2. Note that the ~9 Myr oscillations are present in both compilations in δ^18^O and δ^13^C data with two important features: (1) a possible ~9 Myr coupling between climate and carbon cycle, especially during icehouse (the past ~34 Ma), (2) a strong ~9 Myr decoupling between them within the interval from 65 to 50 Ma: while the δ^13^C registers the two strongest ~9 Myr cycles Cb7 and Cb8, the δ^18^O has only one oscillation (see Fig. S2). In addition, Zachos et al.’s (2001, 2008) compilation (Fig. S1a,b) documents well the 36 Myr oscillations.

Figures S3 and S4 illustrate long-period (> 1 Myr) astronomical cyclicities. Figure S3 shows power spectra of astronomical variations over different time intervals, and considering different astronomical parameters (only eccentricity, only obliquity, and combined eccentricity and obliquity). This procedure points out the origin of each astronomical period (peak). Figure S4 shows amplitude spectrograms of astronomical variations to highlight the ~9 Myr modulation cycles.

**
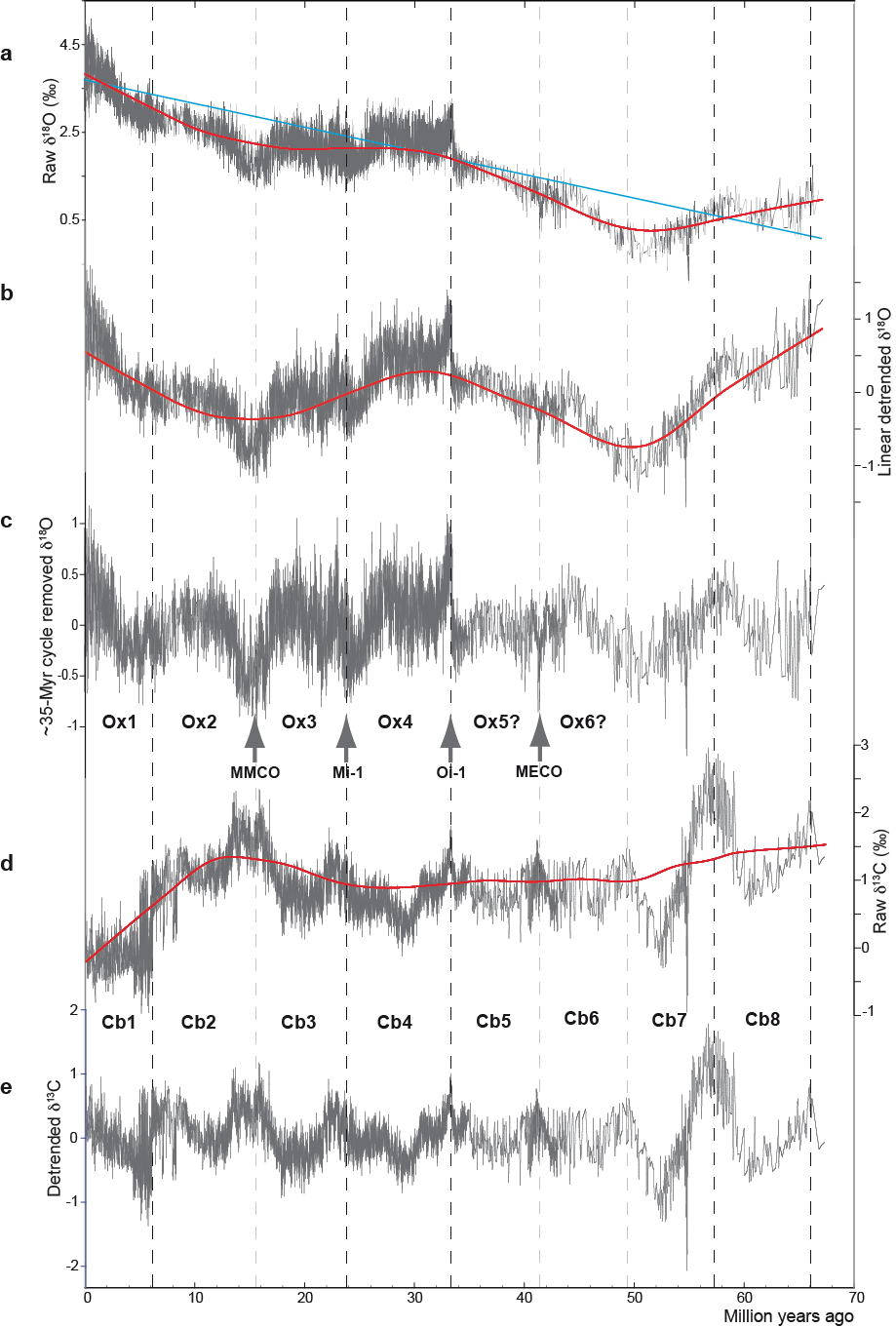
**

**Supplementary Figure S1**: Correlation of Cenozoic benthic foraminifera δ^18^O and δ^13^C variations at the scale of ~9 Myr cyclicity. δ^18^O and δ^13^C data are from Zachos et al. (2001, 2008) revised in Boulila et al. (2012). **(a)** Raw δ^18^O, linear trend and a 25% weighted average of the series are also shown. **(b)** Linear-detrended δ^18^O, a 25% weighted average of the linear-detrended series and the strong ~36 Myr cycle are shown. **(c)** Weightened δ^18^O data (i.e., ~36 Myr cycle removed). Ox1, Ox2, Ox3 and Ox4 are ~9 Myr δ^18^O variations correlated to Cb1, Cb2, Cb3 and Cb4 δ^13^C variations. Question mark at Ox5 and Ox6 indicates uncertain ~9 Myr cycles. Oi-1 and Mi-1 glacial events, MMCO (Mid-Miocene Climatic Optimum), and MECO (Mid-Eocene Climatic Optimum) are also shown. **(d)** Raw δ^13^C, a 25% weighted average of the series is shown. Cb1 to Cb8 represent the interpreted ~9 Myr cycles related to eccentricity modulation cycles as in Boulila et al. (2012). **(e)** Detrended δ^13^C (a 25% weighted average of the series in ‘d’ is removed).


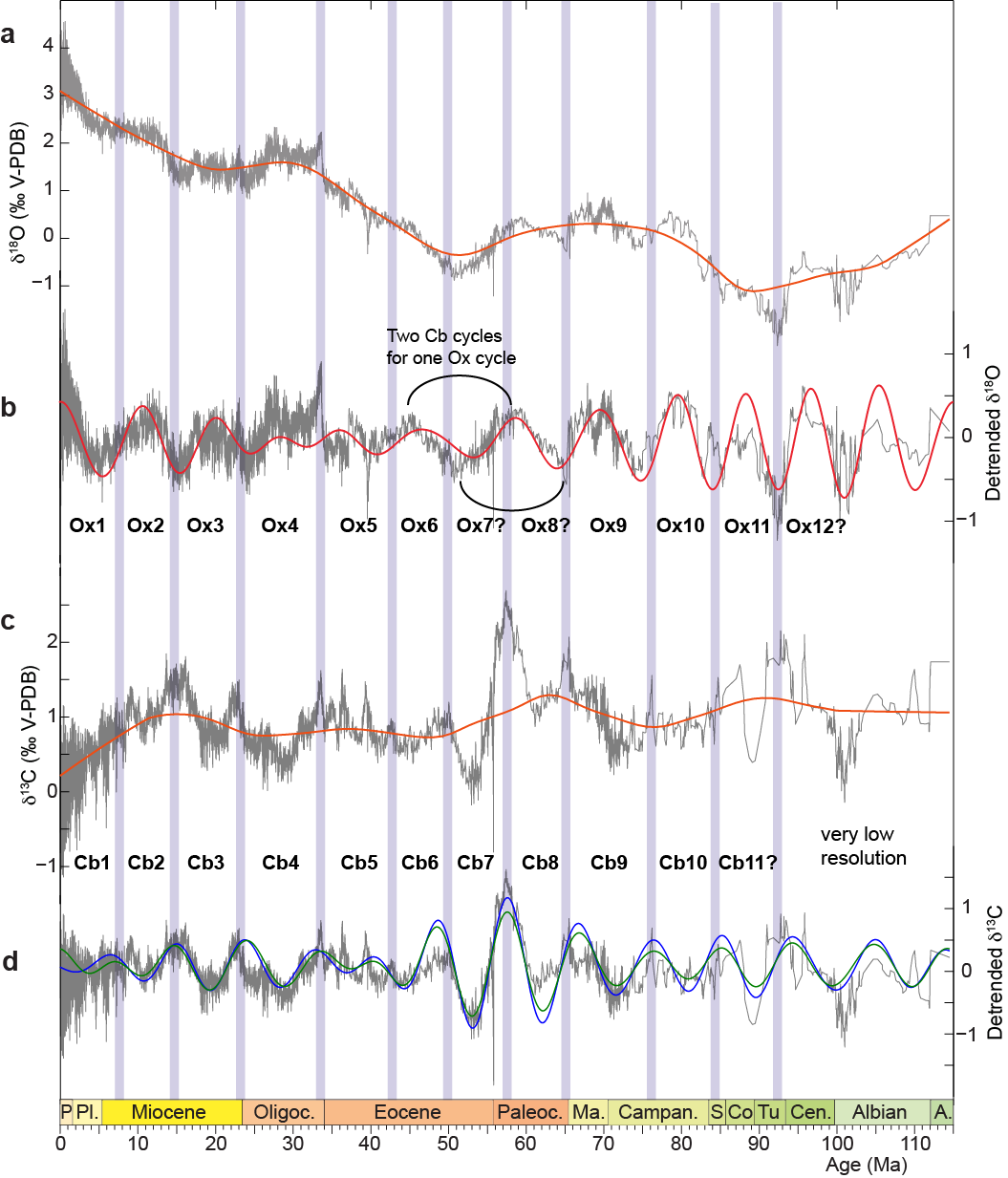


**Supplementary Figure S2**: Correlation of Cenozoic-middle Cretaceous benthic foraminifera δ^18^O and δ^13^C at the scale of ~9 Myr cyclicity. δ^18^O and δ^13^C data are from Cramer et al. (2009) for the Cenozoic and Friedrich et al. (2012) for the Cretaceous. **(a)** Smoothed δ^18^O with 5 point moving average, 15% weighted average of the series is shown. **(b)** Detrended δ^18^O, the 15% weighted average shown in ‘a’ is removed, bandpass (0.11 ±0.03 cycles/Myr) filter output is also shown. **(c)** Smoothed δ^13^C with 5 point moving average, 20% weighted average of the series is shown. **(d)** Detrended δ^13^C, the 20% weighted average shown in ‘c’ is removed, bandpass (0.11 ±0.03 cycles/Myr) filter output is also shown.

Cb1 to Cb11 are interpreted ~9 Myr δ^13^C cycles (Cb1 to Cb8 are as in Boulila et al., 2012). Question mark in Cb11 indicates unconstrained ~9 Myr δ^13^C cycle because of the low resolution. Ox1 to Ox12 in ‘b’ are equivalent ~9 Myr δ^13^O cycles. Note that Ox7 and Ox8 are actually not 9 Myr oscillations, but constitute only one oscillation. I used this terminology for correlation with already recognized δ^13^C cycles (Boulila et al., 2012). This mismatch between ~9 Myr δ^13^O and δ^13^C cycles is indicative of a decoupling between climate and carbone cyle during this extreme greenhouse episode.


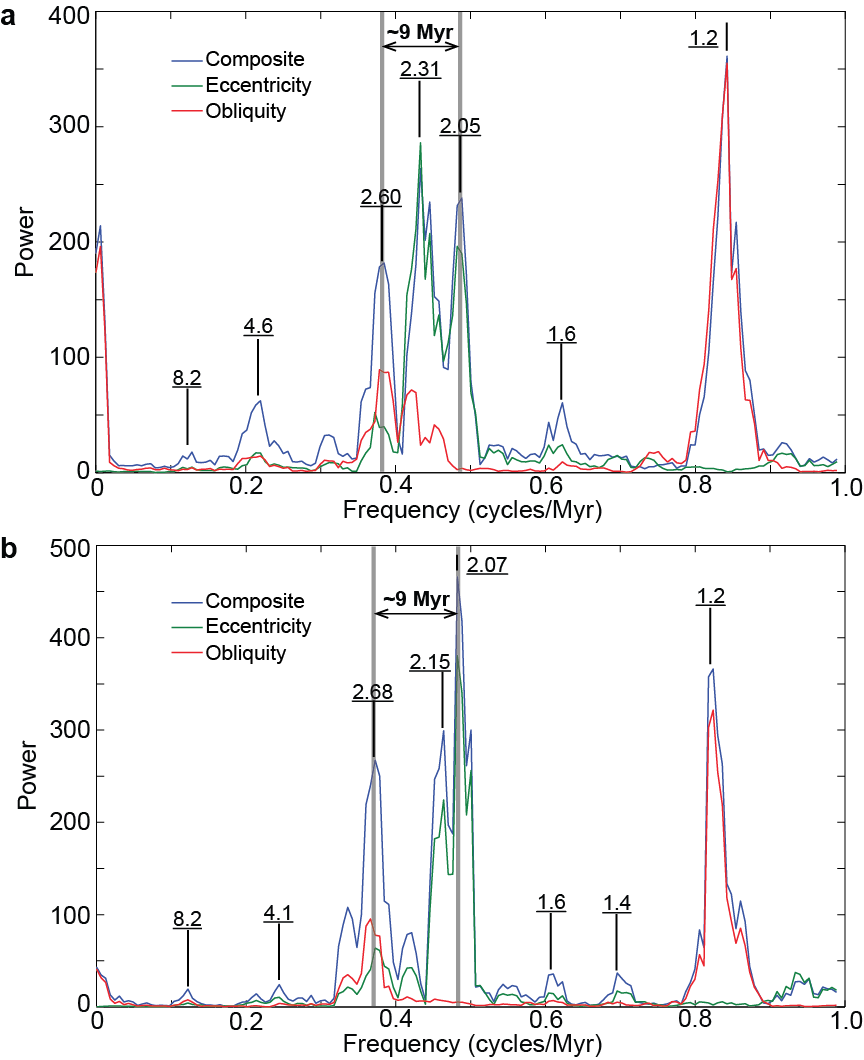


**Supplementary Figure S3**: Power spectra of astronomical variations. **(a)** Spectra of interval from 0 to 150 Ma. **(b)** Spectra of the interval from 100 to 240 Ma. The ~9 Myr period results from the interference of 2.05 Myr vs 2.60 Myr in ‘a’ and 2.07 Myr vs 2.68 Myr in ‘b’ (see also Fig. S4 below). This is a strong evidence for the presence of such modulation throughout all the modelled astronomical variations. The peak of 8.2 Myr is also present in the composite signal, but better within the interval from 100 to 240 Ma. This peak corresponds likely to the ~9 Myr modulation.


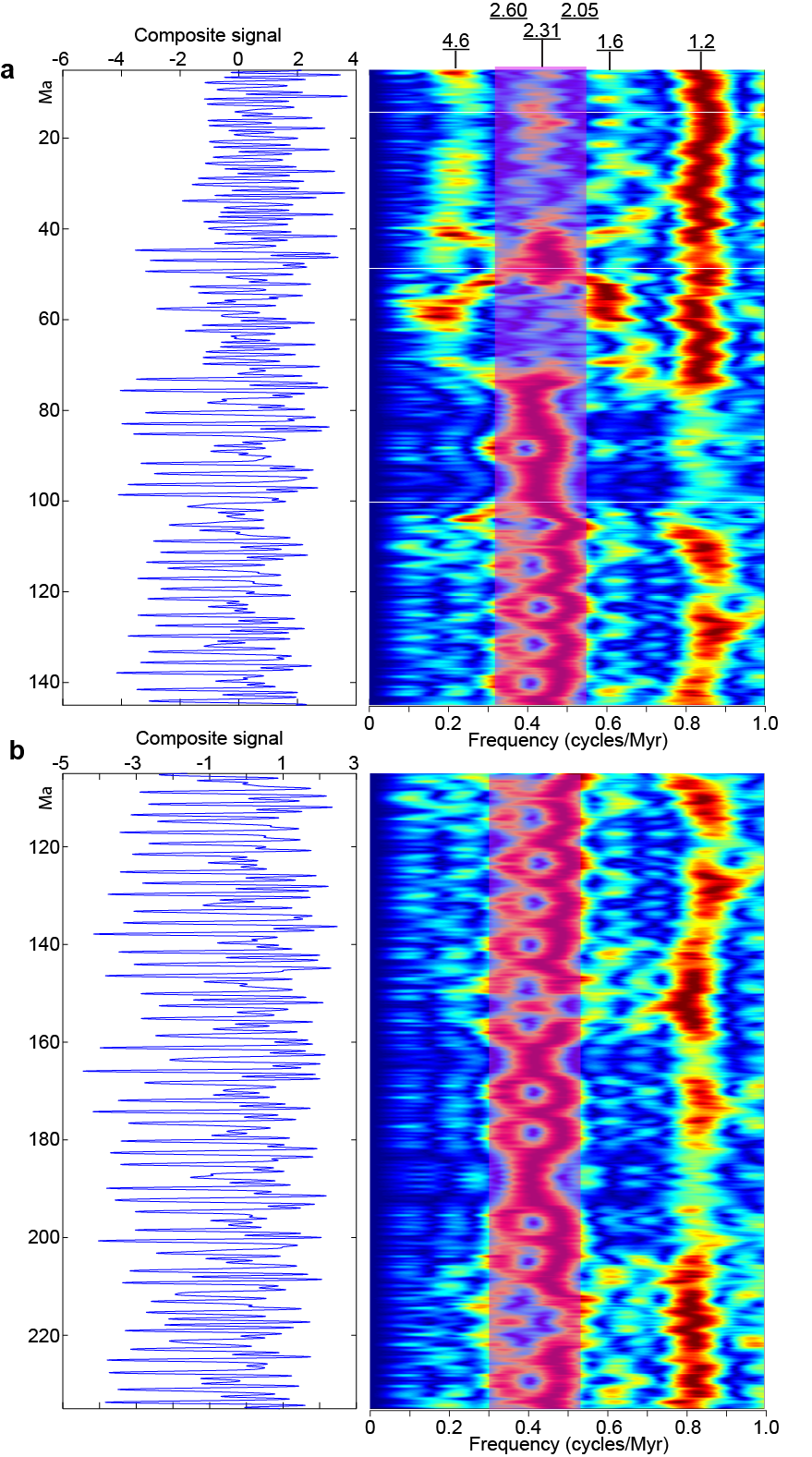


**Supplementary Figure S4**: Amplitude spectrograms of composite astronomical signals to highlight the ~9 Myr modulation cycles. **(a)** Interval from 0 to 150 Ma. **(b)** Interval from 100 to 240 Ma. The shaded area indicates the modulation of the 2 and 2.6 Myr cycles by the ~9 Myr cyclicity.
